# A Case of Radiation Recall Dermatitis of Scalp in Acute Lymphoblastic Leukemia After Prophylactic Cranial Radiotherapy

**DOI:** 10.7759/cureus.1671

**Published:** 2017-09-10

**Authors:** Ponraj Madasamy, Jogamaya Pattnaik, Murali Paramanandhan, Biswajit Dubashi, Naresh Jadhav, Jagdeep Singh

**Affiliations:** 1 Department of Medical Oncology, RCC, Jipmer, Pondicherry, India

**Keywords:** radiation recall dermatitis, leukemia, prophylactic cranial radiotherapy, all

## Abstract

The radiation recall dermatitis (RRD) phenomenon is defined as the “recalling” of the skin following the administration of drugs; this induces a response or flare-like reaction over the skin that is exposed to radiation. In this case report, a young female developed RRD on Day 18 after the completion of cranial radiotherapy, that is, four days after the restart of the chemotherapy with doxorubicin. It is a self-limiting condition with supportive care as the treatment. When encountered in hematological malignancies, undue treatment breaks can delay definitive treatment and can eventually cause a relapse.

## Introduction

Radiation recall dermatitis (RRD), also called radiation recall, is a phenomenon that is defined by the “recalling” of the skin following the administration of drugs; this induces a response or flare-like reaction over the skin that is exposed to radiation. This phenomenon is well-documented in the medical literature, although the exact cause of the flare is unknown [1].

D’Angio described RRD for the first time in 1959 following the administration of dactinomycin [2]. The most common causative drugs being doxorubicin, paclitaxel, docetaxel, methotrexate, cyclophosphamide, and so on, with previous radiation exposure in response to the administration of certain response-inducing drugs. In this case report, we highlight a case of T-cell acute lymphoblastic leukemia (T-ALL), who was treated with the German multicenter ALL (GMALL) protocol, received prophylactic cranial radiotherapy in between the consolidation phase, and developed radiation recall dermatitis on exposure to doxorubicin two weeks following the completion of radiotherapy.

## Case presentation

**Figure 1:**
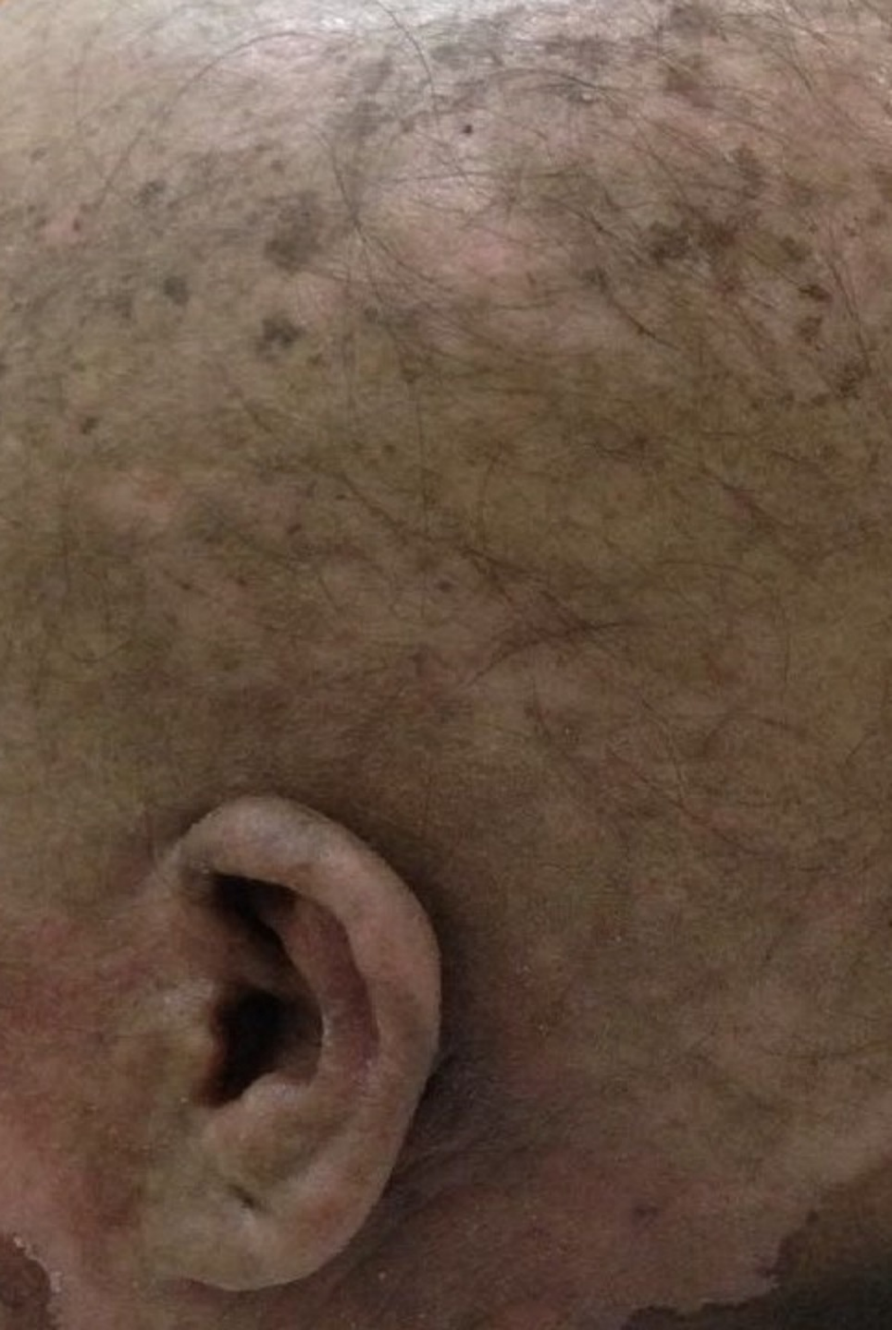
Radiation recall dermatitis of scalp showing epilation of scalp hair with dermatitis over the scalp

 A 30-year-old female patient presented with a history of unexplained fever for three months with associated weight loss. On examination, the patient's vitals were stable and the systemic examination was normal. A complete blood hemogram showed a total count of 30,000/mm^3^, with 80% blasts on the peripheral smear, myeloperoxidase (MPO) negative. Bone marrow and flow cytometry confirmed the diagnosis of T-ALL with normal karyotype and cerebrospinal fluid (CSF) was negative. The patient was started on the GMALL protocol as per the institution protocol. At the end of induction, the patient was in remission. The patient was started on consolidation-1, followed by prophylactic cranial radiotherapy of 24Gy/12 fractions, 2Gy/fraction. Two weeks after the completion of the radiotherapy, consolidation-2 was restarted with doxorubicin. The patient developed a flare reaction over the irradiated area of the scalp and face four days after completion of the chemotherapy, that is, on Day 18 of the completion of radiotherapy. There was loss of hair (epilation) over the scalp with radiation dermatitis over the scalp, as indicated in Figure 1. The chemotherapy protocol was stopped and the patient was started on treatment with topical steroids, supportive care like nonsteroidal anti-inflammatory drugs (NSAIDS), and sunlight exposure. The patient recovered after three weeks and the protocol was restarted, excluding doxorubicin.

## Discussion

Radiation recall dermatitis can be defined as a phenomenon wherein the reinstitution of a cytotoxic drug causes a flare reaction over the previously irradiated area of the skin, ideally after the resolution of the radiation-induced reactions [3]. The RRD can appear anytime within a few days to many years after the completion of radiotherapy. Our case is the first case of RRD reported in India in a hematological malignancy. There are previous studies showing RRD in hematological malignancies [4]. Our case was treated for T-ALL on the GMALL protocol. The patient was restarted on consolidation-2 chemotherapy doxorubicin on Day 14 after the completion of radiotherapy and developed RRD of the scalp on Day 18. As per Cambige and Price, the lag time to develop RRD is greater than seven days [5]. There is no exact timing of restarting chemotherapy after the completion of radiotherapy. It varies from six to 37 days [6]. The timing of chemotherapy may be crucial after the completion of radiotherapy. The patient received 24Gy/12 fractions, 2Gy/fraction. There is no exact association of radiation dose to RRD although there are reports of RRD with doses below 20Gy [5]. The commonest drug causing RRD is doxorubicin [7].

There are many theories postulated for RRD. The most prominent among them is that, during radiotherapy, the cell killing occurs by sublethal and lethal damage. So, a few cells undergoing sublethal damage survive, but the tissue appears to be completely healed. On exposure to the offending drugs, these cells show enhanced response or hyper-responsiveness, leading to RRD. The other hypothesis is that radiotherapy leads to the depletion of tissue stem cells and cytotoxic agents cause a recall reaction in the surviving cells [8]. RRD can also have systemic manifestations involving internal organs, such as lungs, gastrointestinal tract (GIT), head and neck, genitourinary tract, and central nervous system (CNS). In our case, there was only a localized reaction and no systemic involvement.

RRD is a self-limiting phenomenon. Usually, the low-grade flare reactions are self-limiting and resolve on stopping the offending drug. The more severe reactions may require topical, oral steroids, anti-inflammatory drugs, and antihistaminics. Also, supportive care like the application of lotions and creams and avoiding sun exposure should be used. RRD can be managed as per the algorithm proposed by Caloglu et al., where the treatment is based on the severity of the reaction [9]. It usually takes around two weeks to resolve. In our case, the patient had severe RRD, and the protocol was withheld. The patient was started on topical steroids, supportive care like NSAIDS, and avoidance of exposure to the sun. The patient recovered gradually over a period of three weeks and was restarted on the protocol after resolution of the RRD with the omission of doxorubicin.

## Conclusions

RRD is a rare phenomenon that is encountered in clinical practice. The authors would like to conclude that though RRD is not a life-threatening condition, it can always be a challenge to a treating clinician, as undue treatment interruption can cause a relapse in such cases.
